# Murine lung injury caused by *Leptospira interrogans* glycolipoprotein, a specific Na/K-ATPase inhibitor

**DOI:** 10.1186/s12931-014-0093-2

**Published:** 2014-08-14

**Authors:** Cassiano Felippe Gonçalves-de-Albuquerque, Patrícia Burth, Adriana Ribeiro Silva, Isabel Matos Medeiros de Moraes, Flora Magno de Jesus Oliveira, Ricardo Erthal Santelli, Aline Soares Freire, Gerson Silva de Lima, Emilson Domingos da Silva, Camila Ignácio da Silva, Verônica Morandi, Patrícia Torres Bozza, Mauricio Younes-Ibrahim, Hugo Caire de Castro Faria Neto, Mauro Velho de Castro Faria

**Affiliations:** Fundação Oswaldo Cruz, Laboratório de Imunofarmacologia, Instituto Oswaldo Cruz, Pavilhão Ozório de Almeida, Av Brasil 4365, Fiocruz, Rio de Janeiro, RJ CEP 21040 - 900 Brazil; Departamento de Biologia Celular e Molecular, Instituto de Biologia, Universidade Federal Fluminense, Niterói, RJ Brazil; Fundação Oswaldo Cruz, Instituto de Tecnologia em Imunobiológicos-BIO Manguinhos, Departamento de Reativos Para Diagnóstico, Av Brasil 4365 Fiocruz, Rio de Janeiro, RJ CEP 21040 - 900 Brazil; Departamento de Química Analítica, Universidade Federal do Rio de Janeiro, Rio de Janeiro, RJ Brazil; Departamento de Química Analítica, Universidade Federal do Rio de Janeiro, Rio de Janeiro, RJ Brazil; Departamento de Medicina Interna, Faculdade de Ciências Médicas, Universidade do Estado do Rio de Janeiro, Rio de Janeiro, RJ Brazil

**Keywords:** Leptospiral GLP, Na/K-ATPase, Lung injury, Toll-like receptors, Ouabain, MAPK p38

## Abstract

**Background:**

Leptospiral glycolipoprotein (GLP) is a potent and specific Na/K-ATPase inhibitor. Severe pulmonary form of leptospirosis is characterized by edema, inflammation and intra-alveolar hemorrhage having a dismal prognosis. Resolution of edema and inflammation determines the outcome of lung injury. Na/K-ATPase activity is responsible for edema clearance. This enzyme works as a cell receptor that triggers activation of mitogen-activated protein kinase (MAPK) intracellular signaling pathway. Therefore, injection of GLP into lungs induces injury by triggering inflammation.

**Methods:**

We injected GLP and ouabain, into mice lungs and compared their effects. Bronchoalveolar lavage fluid (BALF) was collected for cell and lipid body counting and measurement of protein and lipid mediators (PGE_2_ and LTB_4_). The levels of the IL-6, TNFα, IL-1B and MIP-1α were also quantified. Lung images illustrate the injury and whole-body plethysmography was performed to assay lung function. We used Toll-like receptor 4 (TLR4) knockout mice to evaluate leptospiral GLP-induced lung injury. Na/K-ATPase activity was determined in lung cells by nonradioactive rubidium incorporation. We analyzed MAPK p38 activation in lung and in epithelial and endothelial cells.

**Results:**

Leptospiral GLP and ouabain induced lung edema, cell migration and activation, production of lipid mediators and cytokines and hemorrhage. They induced lung function alterations and inhibited rubidium incorporation. Using TLR4 knockout mice, we showed that the GLP action was not dependent on TLR4 activation. GLP activated of p38 and enhanced cytokine production in cell cultures which was reversed by a selective p38 inhibitor.

**Conclusions:**

GLP and ouabain induced lung injury, as evidenced by increased lung inflammation and hemorrhage. To our knowledge, this is the first report showing GLP induces lung injury. GLP and ouabain are Na/K-ATPase targets, triggering intracellular signaling pathways. We showed p38 activation by GLP-induced lung injury, which was may be linked to Na/K-ATPase inhibition. Lung inflammation induced by GLP was not dependent on TLR4 activation.

## Background

Leptospirosis is a worldwide zoonosis caused by pathogenic spirochetes of the genus *Leptospira*. This disease affects both animals and humans and has veterinary, economic and medical relevance [1]. In tropical countries, epidemic outbreaks occur in the rainy season and after floods [2]. Infection commonly occurs after contact with contaminated soil and water. An intact keratinocyte layer is a barrier against leptospiras [3], but water sprays can facilitate bacterial entry [4]. During the acute phase of the disease, leptospiras are found in the liver and kidneys [5]. Pulmonary involvement in leptospirosis (Weil’s disease) has been reported over the last 20 years and is related to the severity and mortality of the disease [6,7].

Bacterial recognition by a host during leptospirosis is still not completely understood, but the presence of leptospira may be sensed through Toll-like receptors (TLR4 and TLR2) [8,9]. The leptospiral lipopolysaccharide (LPS) differs from those found in other Gram(-) bacteria. In this regard, lipid A, the active component of leptospiral LPS, is structurally and functionally different from that of *E. coli* [10]. The recognition of *L. interrogans* LPS requires CD14 and TLR2, but *L. interrogans* LPS is incapable of inducing intracellular signaling through TLR4 activation [9]. A key protein of the outer leptospiral membrane, the lipoprotein LipL32, is produced during leptospirosis [11]. This protein is highly conserved and found exclusively in pathogenic leptospiras [12]. LipL32 has been shown to activate TLR2 [9] in a Ca^2+^-binding cluster-dependent manner [13].

Another leptospiral component with cytotoxic activity is the glycolipoprotein fraction GLP [14]. The observation that GLP causes a decrease in renal water absorption provides new evidence that this component is an important contributor to the virulence of pathogenic *Leptospira* [15]. Due to their peculiar metabolism, leptospiras are able to store fatty acids [14]. Some of them (e.g., palmitovaccenic and linoleic acids) are associated with GLP [14], while others (e.g., hydroxylauric and palmitic acids) are associated with LPS and lipopolysaccharide-like substance (LLS) [16]. Oleic acid is associated with both LPS and GLP.

We have proposed that nonesterified fatty acids (NEFA) produce toxic effects and are involved in multi-organ failure that is characteristic of Weil’s disease [17]. Supporting those findings, we have demonstrated increased molar ratios of serum NEFA; in particular, the linoleic and oleic acids/albumin molar ratios are increased in severe forms of leptospiral infection [17].

The resolution of pulmonary edema and lung inflammation are important determinants of the outcome of acute respiratory distress syndrome (ARDS) [18]. Resolution of alveolar edema is dependent on the transfer of salt and water across the alveolar epithelium through apically located sodium channels (ENaC) followed by extrusion to the lung interstitium via the basolaterally located Na/K-ATPase [19].

GLP inhibits Na/K-ATPase [20], and oleic acid has been shown to inhibit Na/K-ATPase in the lung in a rabbit model, resulting in a complete block of active sodium transport and enhancement of endothelial permeability [21]. Cardiac glycosides are a large family of clinically relevant, specific Na/K-ATPase inhibitors that have been classically used to treat heart failure [22]. In addition to their classical effects, ouabain induces internalization and lysosomal degradation of Na/K-ATPase [23], triggering intracellular pathways (including MAPK activation) [24] and inducing lung injury [25].

Increased cytokine production correlates with a lethal outcome in leptospirosis patients [26]. IL-6 release seems to play a key role in acute respiratory distress syndrome (ARDS), although its detailed mechanism of action remains unclear [27]. In addition, the infection of guinea pigs with *L. interrogans* serovar Icteroheamorrhagiae leads to increased IL-6 and TNFα mRNA levels in the lung [28]. IL-1β and IL-18 are produced as cytosolic precursors that require secondary proteolytic cleavage, which is dependent on inflammasome activation [29]. The inflammasome consists of several proteins. One of these, NLRP3, is involved in the recognition of bacterial RNA, ATP, uric acid and low intracellular potassium concentrations (which is a consequence of the inhibition of Na/K-ATPase) [30].

In Brazil, the clinical patterns of leptospirosis have changed, and severe cases with pulmonary involvement have been detected [31,32]. Nevertheless, the associated pulmonary distress is not due to extensive bacterial lung colonization [33,34]. Leptospiral components, including GLP, can induce lung injury following their release by bacteria killed during the immune response, as they can ultimately reach the lung.

Oleic acid, an inhibitor of Na/K-ATPase activity [20,21], has been used experimentally to induce lung injury in mice [35]. Intravenously and intratracheally injected oleic acid targets lung Na/K-ATPase *in vivo* [25,36]. In this respect, GLP is a much more specific Na/K-ATPase inhibitor than only oleic acid [20]. Oleic acid-induced lung injury and IL-6 production in the murine lung occur through ERK1/2 activation [36], and as we have shown, Na/K-ATPase is a target for GLP and fatty acids and plays an important role in leptospirosis physiopathology [37]. GLP fatty acid components, including oleic acid, are responsible for the biological effects of GLP [20].

In this study, we compared two specific Na/K-ATPase inhibitors (GLP and ouabain), which were administered through the intra-tracheal route, for their ability to induce lung edema, cell migration and activation, and the production of lipid mediators and cytokines in different mouse strains. Because oleic acid triggers lung injury through MAPK ERK [38], we also investigated if GLP can activate the MAPK pathway.

## Methods

### Animals

We used male mice (25 – 30 g) of the following strains: Swiss Webster (SW) and C57Bl/10 (from the Oswaldo Cruz Foundation breeding unit, Rio de Janeiro, Brazil) and C57Bl/10ScCr (kindly provided by the Federal Fluminense University breeding unit, Rio de Janeiro, Brazil). The animals were housed at 22°C with a 12-h light/dark cycle and free access to food and water.

### Ethical statement

The Animal Welfare Committee of the Oswaldo Cruz Foundation approved all the experiments under license numbers 002-08 and LW36/10 (CEUA/FIOCRUZ).

### Reagents

Ouabain (purity > 99%) and *E. coli* LPS were obtained from Sigma-Aldrich, St. Louis, MO. All other reagents were of the highest purity grade.

### Preparation of the GLP fraction

GLP was prepared from *Leptospira interrogans* serovar Copenhageni strain Fiocruz L1-130 as previously described [14]. *L. interrogans* was grown at 28°C in EMJH medium (Bio-Rad) without agitation. At the end of the exponential phase, the bacteria were pelleted at 9000 × *g* in an endotoxin-free, 50-mL polypropylene tube and frozen at -80°C. The pellet was resuspended in endotoxin-free 0.01 M Tris/HCl (pH 7.4), lysed at 4-8°C for 48 h by agitating with glass beads and then centrifuged at 20,000 × *g* for 30 min at 18°C. The supernatant was treated with RNase and DNase (50 μg/mL each for 3 h at 37°C) and then dialyzed for 24 h at 4°C against 0.1 M Tris/HCl buffer (pH 7.4). After acidification to pH 3.7 with 1 M acetic acid at 4°C, GLP was sedimented by ultracentrifugation (4000 × *g*, 30 min, 4°C), washed twice with 0.1 M acetic acid and lyophilized. The lyophilized GLP was kept frozen at -20°C. At the time of usage, the lyophilized powder was suspended in sterile saline, and 50 μL of the solution, containing 12 μg of GLP protein, was injected into each animal. The Bradford method was used to determine the protein concentration of the GLP preparation [39]. The preparation (6 μg of GLP protein) retained its inhibitory properties, inhibiting approximately 50% the activity of a standard Na/K-ATPase preparation according to our previous work [20]. This means that the GLP inhibitory properties were tested *in vitro* prior to its injection into mice lung.

### Intra-tracheal administration of GLP, ouabain or Gram(-) LPS

Each mouse was anesthetized with isoflurane, and an incision was made above the thyroid region to expose the trachea. Then, using a syringe, 50 μL of the following doses were instilled into the tracheas of different groups of mice: ouabain (0.075 μmol/animal), GLP (12 μg of GLP protein/animal), Gram(-) *E. coli* LPS (500 ng/animal) and, for the control groups, 50 μL of sterile saline. Ouabain was injected into SW mice, *E. coli* LPS was injected into C57Bl/10 and C57Bl/10ScCr, and GLP was injected into all mouse strains.

### Total and differential cell analysis and total protein quantification in bronchoalveolar lavage fluid (BALF)

After euthanizing the mice in a CO_2_ chamber, the tracheas were isolated by blunt dissection, and three 1.0-mL aliquots of PBS were sequentially instilled into each animal through a small-caliber tube inserted into the airway. After gentle aspiration, 1 mL of fluid was recovered per instillation/aspiration cycle. The aliquots were pooled, for a total of approximately 3 mL of bronchoalveolar lavage fluid per mouse. Total leukocyte counts were performed using microscopy and Neubauer chambers after diluting the BALF samples in Türk solution (2% acetic acid). The differential leukocyte counts of cytocentrifuged smears stained by the May-Grunwald-Giemsa method were determined. The total protein in the BALF supernatants was determined using the Micron BCA Kit method (Pierce) according to the manufacturer’s instructions.

### Lipid body staining and quantification

While still moist, the leukocytes on the CytoSpin slides were fixed in 3.7% formaldehyde in Ca^2+^- and Mg^2+^-free Hank’s buffered salt solution (HBSS; pH 7.4) and stained with 1.5% OsO4 [40]. The lipid bodies in 50 consecutively scanned leukocytes were enumerated by microscopy.

### Cytokine/chemokine measurement assays

The IL-6, CCL3/MIP-1α, TNFα and IL1β concentrations in the cell free-BALF supernatants were measured using ELISA kits according to the manufacturer’s instructions (Duo Set, R&D Systems, Minneapolis, USA).

### PGE2 and LTB4 assays

The concentrations of LTB_4_ and PGE_2_ in the BALF supernatants were assayed using enzyme immunoassay (EIA) kits according to the manufacturer’s instructions (Cayman Chemical, Ann Arbor, MI, USA).

### Measurement of airway function

The airway function in individual unrestrained animals was evaluated 24 h after challenge by barometric plethysmography using a whole body plethysmograph (WBP, Buxco, Troy, NY) as previously described [41].

### Morphological studies

Twenty-four hours after challenge with leptospiral GLP, *E. coli* LPS, ouabain or saline, the animals were euthanized in a CO_2_ chamber, and the lungs were removed. For microscopic studies, the lungs were fixed in 10% neutral-buffered formalin, embedded in paraffin, sectioned at 4 μm and stained with hematoxylin and eosin.

### Isolation of human endothelial cells

Primary human umbilical vein endothelial cells (HUVECs) were isolated as previously described [42,43] and grown in 199 medium (M-199, Sigma) supplemented with 15 mM HEPES, antibiotics (100 U penicillin/mL, 100 mg streptomycin/mL), 2 mM L-glutamine, and 20% (v/v) fetal calf serum (FCS, Cultilab complete medium). The cells were used at the first or second passages only, and subcultures were obtained by treatment of confluent cultures with 0.025% (w/v) trypsin/0.2% (w/v) EDTA in PBS. Cell viability was assessed with Trypan blue, and it remained above 95%.

### Epithelial cell culture experiments

A549 lung epithelial cells were kindly provided by Dr. Cristina Plotkowski (from the Rio de Janeiro State University, Rio de Janeiro, RJ, Brazil) and were maintained in complete DMEM/F12 (Hyclone) medium (containing 2% fetal bovine serum, 1% penicillin, and 100 UI/mL streptomycin). A day before the experiment, the cells were treated with trypsin (0.025%), centrifuged at 4°C and 400 × g for 10 min, resuspended in the complete medium, and incubated at 37°C in 5% CO_2_ in 24-well plates (300,000 cells per well). The cells were then washed with PBS 30 min after the stimulus, lysed with lysis buffer (10 mM Tris pH 8.0, 150 mM NaCl, 1% Triton) containing protease inhibitors (Complete Protease Inhibitor Cocktail Tablets from Roche), and stored at -20°C.

### Lung tissue experiments

Animals were anesthetized with ketamine and xylazine and then perfused with 20 mL of 20 mM ethylenediaminetetraacetic acid (EDTA) pH 7.4 through the right cardiac ventricle. Then, the lung tissues were cut into small pieces and homogenized at 4°C in a homogenizer using the lysis buffer containing protease inhibitors.

### Evaluation of p38 activation in cultured cells and lung tissues

Cell suspensions and lung lysates in the electrophoresis sample buffer were heated at 100°C for 5 min and run in 10% polyacrylamide gels (PAGE-SDS). After transfer of the proteins to nitrocellulose membranes at 15 V for 60 min (Biorad semidry system), the membranes were incubated with a blocking solution followed by incubation with a monoclonal antibody against phosphorylated p38 (Cell Signaling; 1:1000 dilution) and then with a peroxidase-conjugated anti-mouse antibody (Pierce; 1:10,000). Detection was performed by utilizing the “Super Signal Chemiluminescence” kit (Pierce) and then exposing the membrane to an autoradiograph film (Kodak MR Biomax). Membranes containing proteins were stripped, blocked again, and incubated with monoclonal antibodies to total p38 (Cell Signaling; 1:1000) or glycerol-3-phosphate dehydrogenase (GAPDH) followed by treatment with an anti-mouse antibody conjugated to peroxidase. After digitalized and analysis by size and intensity using the Image Master 2D Elite 4.01 equipment, the bands were compared to those of the controls and normalized against total p38 or GAPDH. The expression results are in folds over the controls.

### Treatment with a MAPK p38 phosphorylation inhibitor

The p38 phosphorylation selective inhibitor SB203580 (4-(4-fluorophenyl)-2-(4-methylsulfinylphenyl)-5-(4- pyridyl)imidazole) at a concentration of 10 μM was incubated with A549 cells 30 min before GLP stimuli. The SB203580 was previously dissolved in dimethylsulphoxide (DMSO) and diluted with PBS when used.

### Na/K ATPase assay in HUVECs based on Rb^+^ incorporation

*E.coli* LPS, ouabain and leptospiral GLP were diluted in KCl free-Hank’s solution containing non-radioactive Rb^+^ and incubated with cells for 30 min at 37°C in 5% CO_2_ atmosphere. The culture was washed with cold PBS and cells were lysed with 600 μL of SDS 0.25%. Samples were centrifuged 10000 × *g* [44] and Rb^+^ was quantified by inductively coupled plasma optical emission spectrometry (ICP-OES) using an Ultima 2 apparatus with Mira Mist Nebulizer and spray chamber (Jobin Yvon, Longjumeau, France). A rubidium nitrate standard (Ultra Scientific, EUA) was used to construct the calibration curve. Results were expressed in μmol of Rb^+^ incorporated per 30 min per 3 × 10^5^ cells.

### Statistical analysis

Results were expressed as the mean ± standard error of the mean (SEM) and analyzed by one-way ANOVA followed by the Newman-Keuls-Student posttest to compare all columns. Differences were considered significant when p < 0.05.

## Results

**Figure 1:**
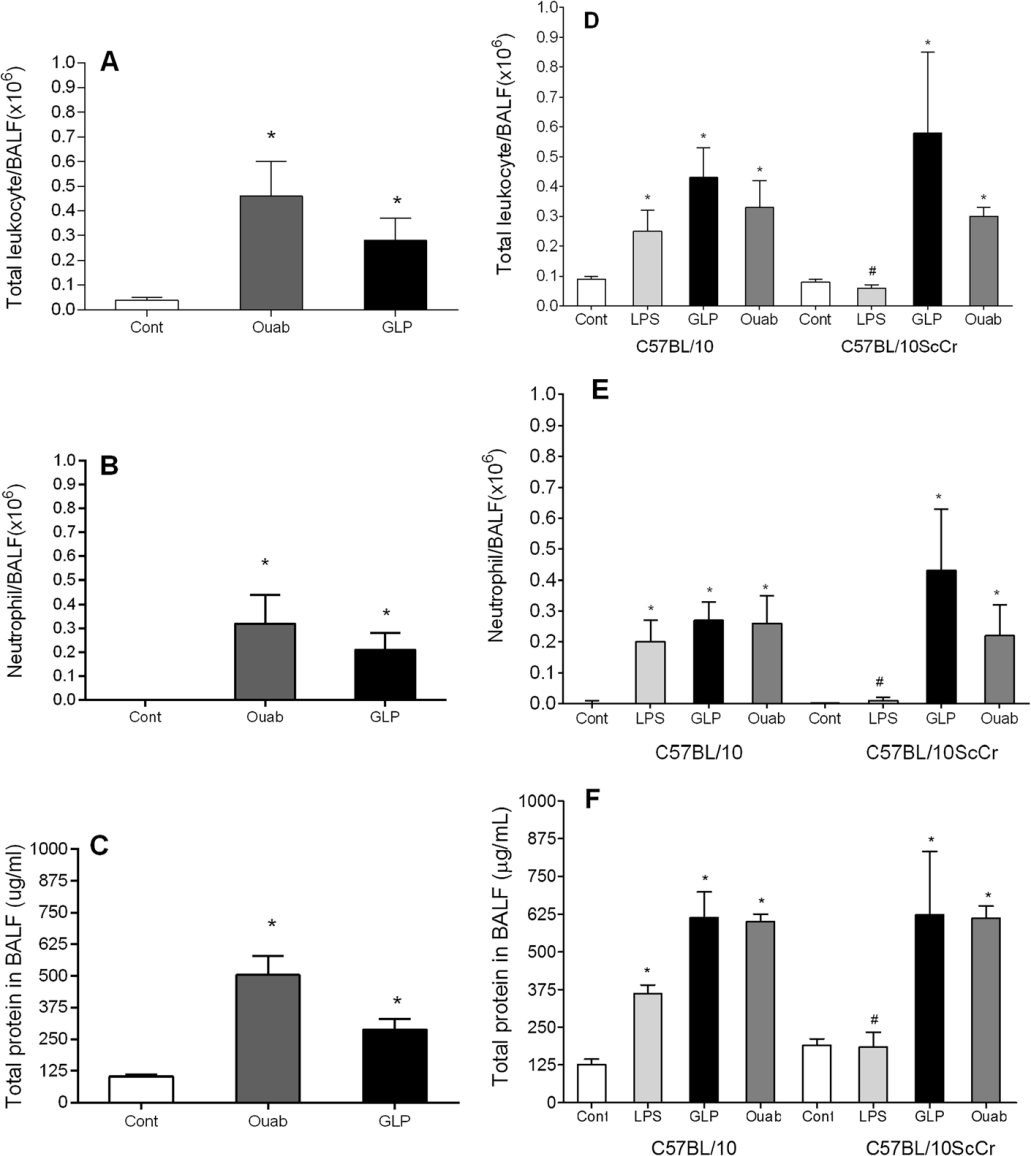
**Cell migration and protein extravasation in BALF of SW mice 24 h after intra-tracheal challenge with ouabain (0.075 μmol/animal) or GLP (12 μg of GLP protein/animal). (A)** Total cells, **(B)** neutrophils, and **(C)** total protein in BALF supernatant. Controls received 50 μL of sterile saline. Cell migration and edema formation in lungs of C57BL/10 and C57BL/10ScCr (TLR4-deficient) mice 24 h after intra-tracheal challenge with LPS (500 ng/animal) or GLP (12 μg of GLP protein/animal). Controls received 50 μL of sterile saline. Controls (white bars), LPS-treated (gray bars), and GLP-treated (black bars). **(D)** Total cells, **(E)** neutrophils and **(F)** total protein in BALF supernatants. The results are the means ± SEM of at least 8 animals. *P < 0.05 compared to the control group; ^#^P < 0.05 when both strains were compared.

GLP (12 μg of protein/animal) and ouabain (0.075 μmol/animal) injected intratracheally induced cell accumulation in BALF, as demonstrated by the increased number of neutrophils (Figure 1A and B). The total protein concentration of BALF supernatants was also increased after GLP administration (Figure 1C), indicating increased vascular permeability and edema formation.

As the activation of TLR4 protects mice against lethal leptospiral infection [45], we compared the effects of leptospiral GLP and *E. coli* LPS challenges in C57BL10/ScCr mice, which possess a null mutation for TLR4 and are therefore resistant to high doses of LPS [46]. The wild-type controls showed a typical response to LPS, with increased cell migration and higher total protein content in BALF, while, as expected, the C57BL10/ScCr mice did not respond to LPS. GLP and ouabain not only induced cell accumulation in BALF but also augmented the total protein in both C57BL10/ScCr and wild type control animals (Figure 1D, E and F), thus excluding the possibility of LPS contamination of our GLP preparation.

**Figure 2:**
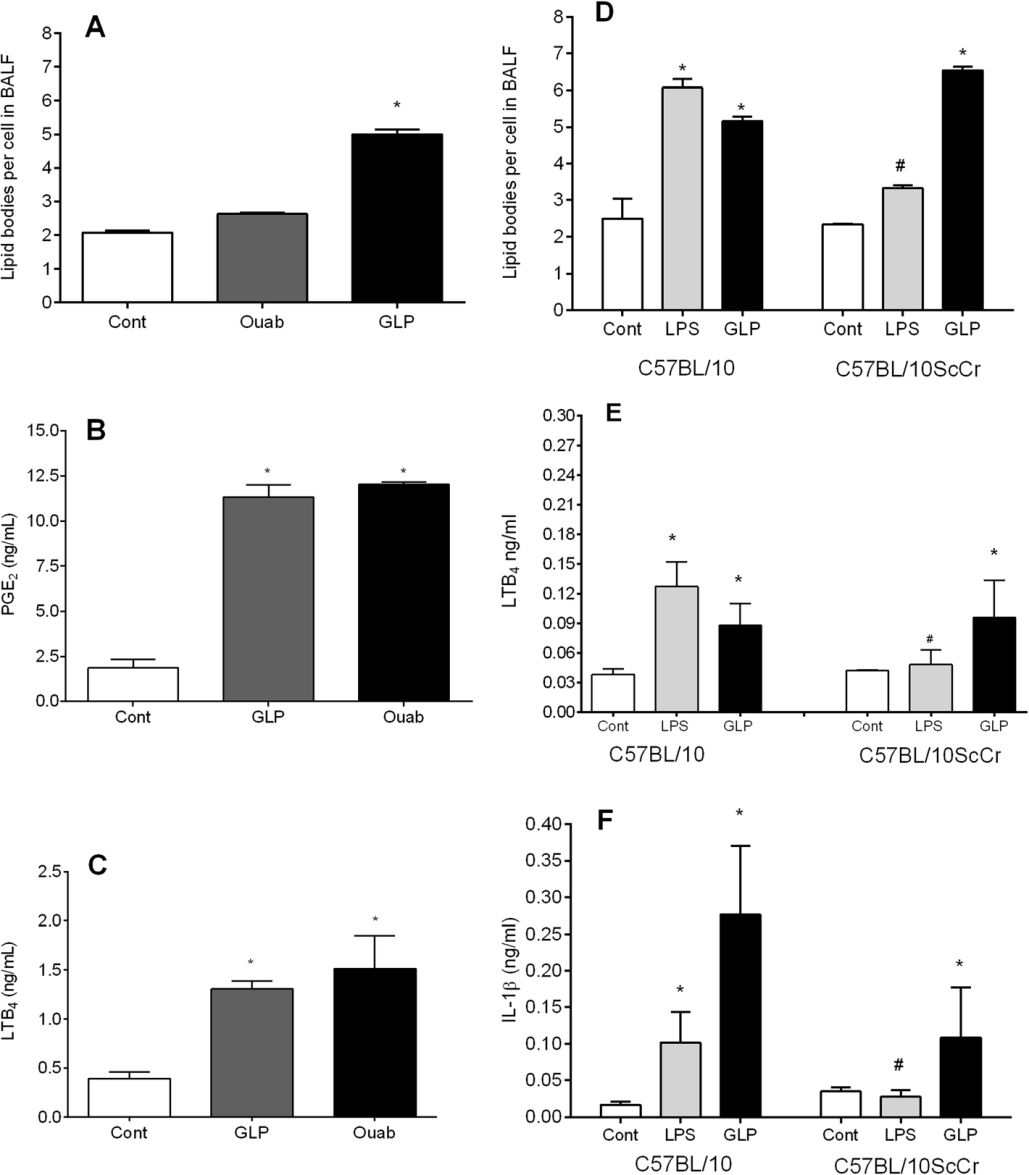
**Lipis body formation and lipid mediators production in BALF of SW mice 24 h after intra-tracheal challenge with ouabain (0.075 μmol/animal) or GLP (12 μg of GLP protein/animal).** Lipid body numbers **(A)**, PGE_2_ concentrations **(B)** and LTB_4_ concentrations **(C)** in BALF of SW mice 24 h after intra-tracheal challenge with ouabain (0.075 μmol/animal) or GLP (12 μg of GLP protein/animal). Controls received 50 μL of sterile saline. Number of leukocyte lipid bodies and the production of LTB_4_ and IL-1β in BALF supernatants of C57BL/10 and C57BL/10ScCr mice 24 h after intra-tracheal challenge with LPS (500 ng/animal) or GLP (12 μg of GLP protein/animal). Controls received 50 μL of sterile saline. Controls (white bars), LPS-treated (gray bars), and GLP-treated (black bars). **(D)** Lipid bodies, **(E)** LTB_4_ and **(F)** IL-1β. The results are the means ± SEM of 6 to 8 animals per group. *P < 0.05 compared to the control group; ^#^P < 0.05 when both strains were compared.

**Figure 3:**
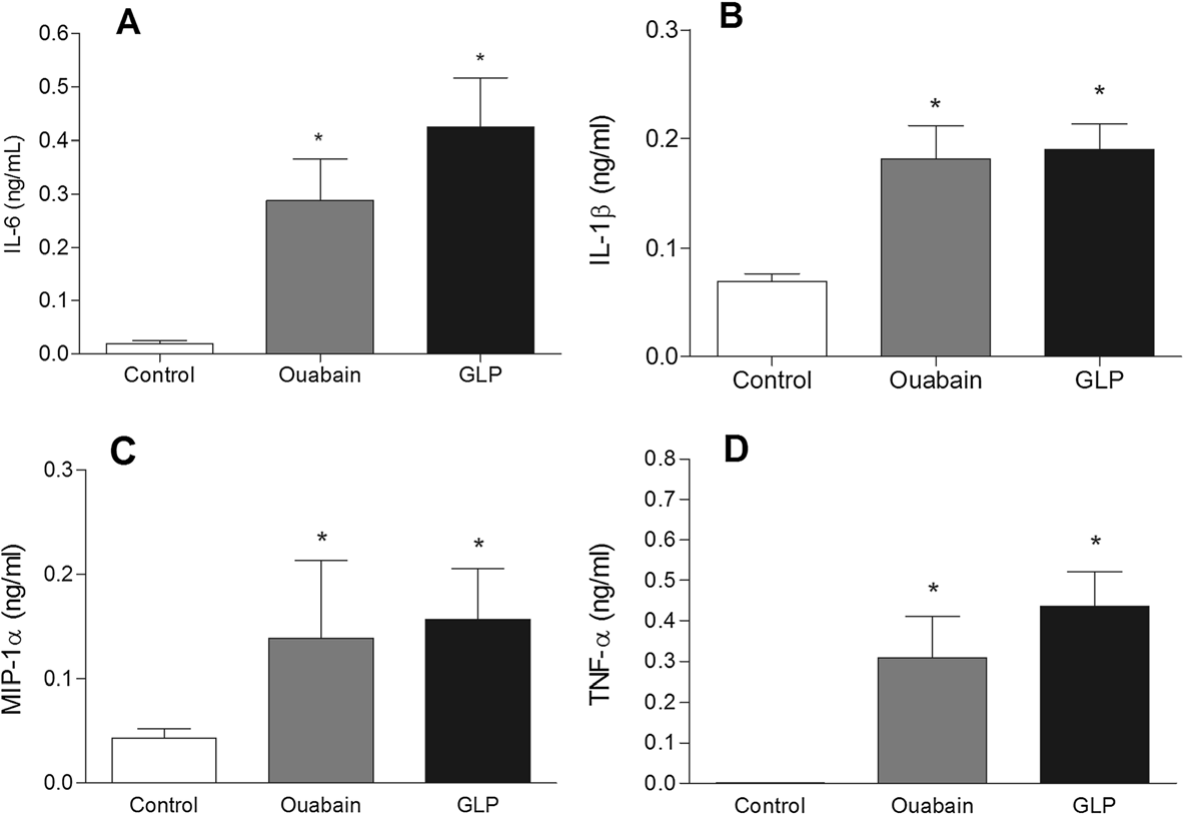
**Production of inflammatory cytokines in BALF supernatants of SW mice 24 h after intra-tracheal challenge with ouabain (0.075 μmol/animal) or GLP (12 μg of GLP protein/animal). (A)** IL-6, **(B)** IL-1β, **(C)** MIP-1α, and **(D)** TNF-α. Controls received 50 μL of sterile saline. The results are the means ± SEM of at least 8 animals.

BALF leukocytes showed signs of activation because lipid body numbers were markedly augmented 24 h after GLP administration (Figure 2A). The concentrations of the lipid mediators PGE_2_ (Figure 2B) and LTB_4_ (Figure 2C) were significantly increased in the BALF supernatants after GLP or ouabain challenge. In addition to inflammatory lipid mediators, we also observed increased cytokine concentrations (IL-6, IL-1β, MIP-1α and TNFα) in BALF supernatants 24 h after administration of GLP or ouabain (Figure 3A, B, C and D, respectively).

As TLR4 is an important leptospiral sensor, we further investigated other inflammatory parameters, such as lipid body formation and the production of LTB_4_ and IL-1β, in TLR4-deficient mice. GLP-induced lipid body formation was similar in wild-type and C57BL10/ScCr mice (Figure 1D). The same pattern was observed for the IL-1β and LTB_4_ levels in BALF supernatants (Figure 1B and C). Again, as expected, LPS did not induce lipid body formation or the production of IL-1β and LTB_4_ in C57BL10/ScCr mice.

**Figure 4:**
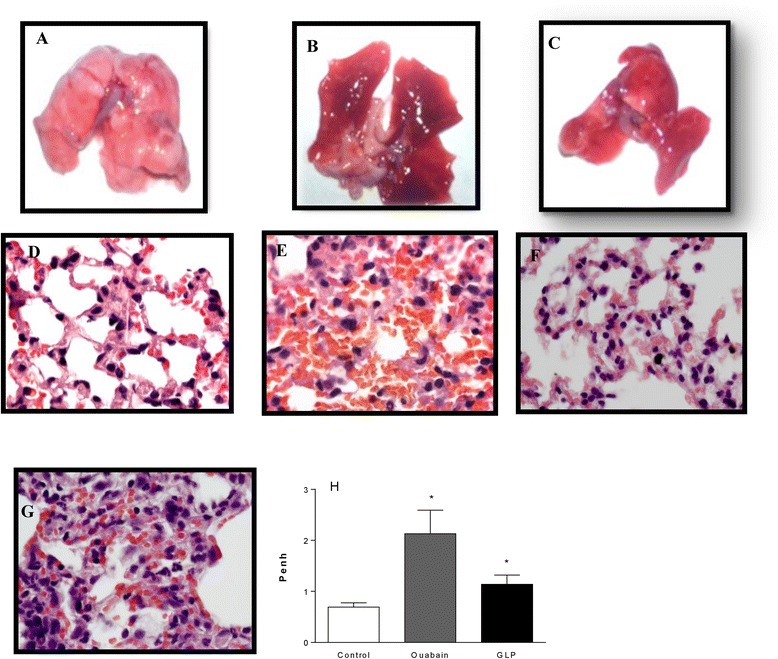
**Representative macro and microphotographs of SW mouse lungs 24 h after intra-tracheal challenge with ouabain (0.075 μmol/animal), GLP (12 μg of GLP protein/animal) or ouabain plus GLP.** Macroscopic photos of mouse lungs challenged with **(A)** sterile saline (control), **(B)** ouabain or **(C)** GLP. Photomicrographs of the same lungs, showing intact alveolar structures in the control group **(D)** and hemorrhagic sites in the lungs from mice challenged with leptospiral GLP **(E)**, ouabain **(F)** or GLP plus ouabain (GLP + ouabain) **(G)**. Plethysmographic analysis of lung function **(H)**, where the enhanced pause (Penh) was used to represent airway resistance. The plethysmographic results are represented as the mean ± SEM of 7 to 15 animals. *P < 0.05.

The lung is a key target in leptospirosis; ARDS patients frequently present hemorrhage, which can be fatal [32], and leptospirosis-susceptible animals present lung hemorrhage [47]. Macroscopic lung evaluation (Figure 4B and C) clearly showed intense hemorrhage 24 h after GLP or ouabain administration compared to controls (Figure 4A). Microscopic analyses of the lungs confirmed intense alveolar hemorrhage in GLP- (Figure 4E), ouabain- (Figure 4F) and GLP plus ouabain-treated animals (Figure 4G) compared to control animals (Figure 4D). All stimuli induced structural damage in the lung, with inflammatory cell infiltration and focused hemorrhage, characterizing a direct lung insult. Ouabain induced a less-intense hemorrhage, and animals injected with GLP plus ouabain presented high mortality rates immediately after injection (data not shown). Functional analysis by lung plethysmography (bar graph, Figure 4H) revealed altered pulmonary function after challenge with ouabain or GLP.

**Figure 5:**
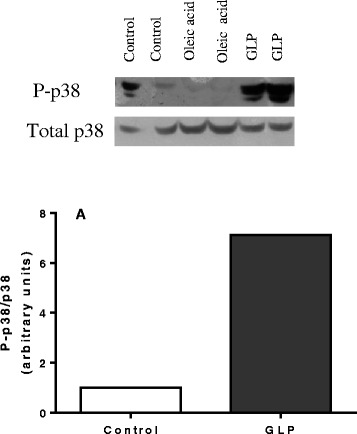
**GLP-induced p38 phosphorylation in the lung tissue.** Lungs were removed from SW animals 4 h after the GLP challenge. The graphs represent densitometry analysis of the phosphorylated p38 and total p38 bands **(A)**, as detailed in the Methods section. The bars represent the medians of the two animals. Oleic acid, a constituent of GLP did not activate p38.

**Figure 6:**
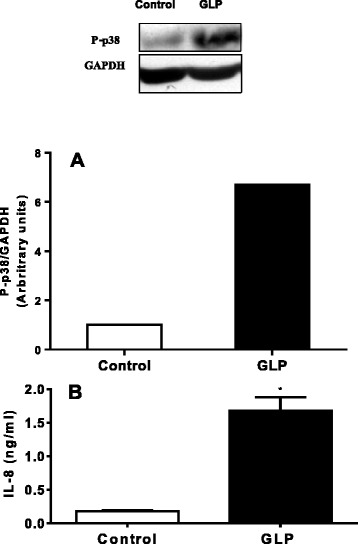
**GLP-induced p38 phosphorylation and IL-8 production in HUVECs.** Cultured cell lysates were prepared after incubation with GLP for 30 min or for up to 24 h to measure the IL-8 level **(B)** the in the supernatant. The graphs represent densitometry analysis of phosphorylated p38 and total p38 bands **(A)**, as detailed in the Methods section.

Na/K-ATPase is a signal transducer that interacts with different signaling proteins to form a complex called the signalosome [48]. One pathway involves activation of the Ras-Raf-MAPK cascade [49], which ultimately activates ERK, c-jun kinase (JNK) and p38. In this regard, we evaluated p38 activation in the lung, lung epithelial cells and human endothelial cells. GLP induced p38 activation, increasing its phosphorylation in lung (Figure 5A), which was different from the effect of oleic acid. Oleic acid activates the ERK pathway [38]. GLP induced p38 phosphorylation (6.7×) and the production of the IL-8 chemokine in human endothelial cells (Figure 6A and B) as well as lung inflammation, demonstrating that the intra-tracheal administration of leptospiral GLP causes lung injury. Based on our previous *in vitro* report showing GLP as a Na/K-ATPase inhibitor [20,50] and the results of the present work, we suggest that this enzyme is a target for GLP *in vivo*.

**Figure 7:**
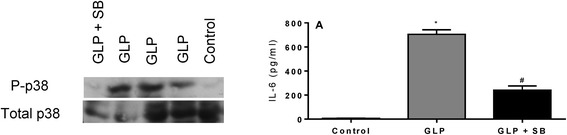
**Blocking p38 activation by treatment with the selective inhibitor SB203580 decreases GLP-induced p38 phosphorylation and IL-6 production.** Cultured cell lysates were prepared after incubation with SB203580 and GLP for 30 min, as detailed in the Methods section. Cultured cell were incubated with GLP for up to 24 h to measure the IL-6 level in the supernatant **(A)**. *P < 0.05 compared to the control group; ^#^P < 0.05 compared to the GLP group.

Finally, GLP induced p38 phosphorylation and IL-6 production in lung epithelial cells, and this effect was reversed by the selective p38 inhibitor SB203580 (Figure 7A), which addresses a key role of p38 in GLP-induced lung inflammation.

**Figure 8:**
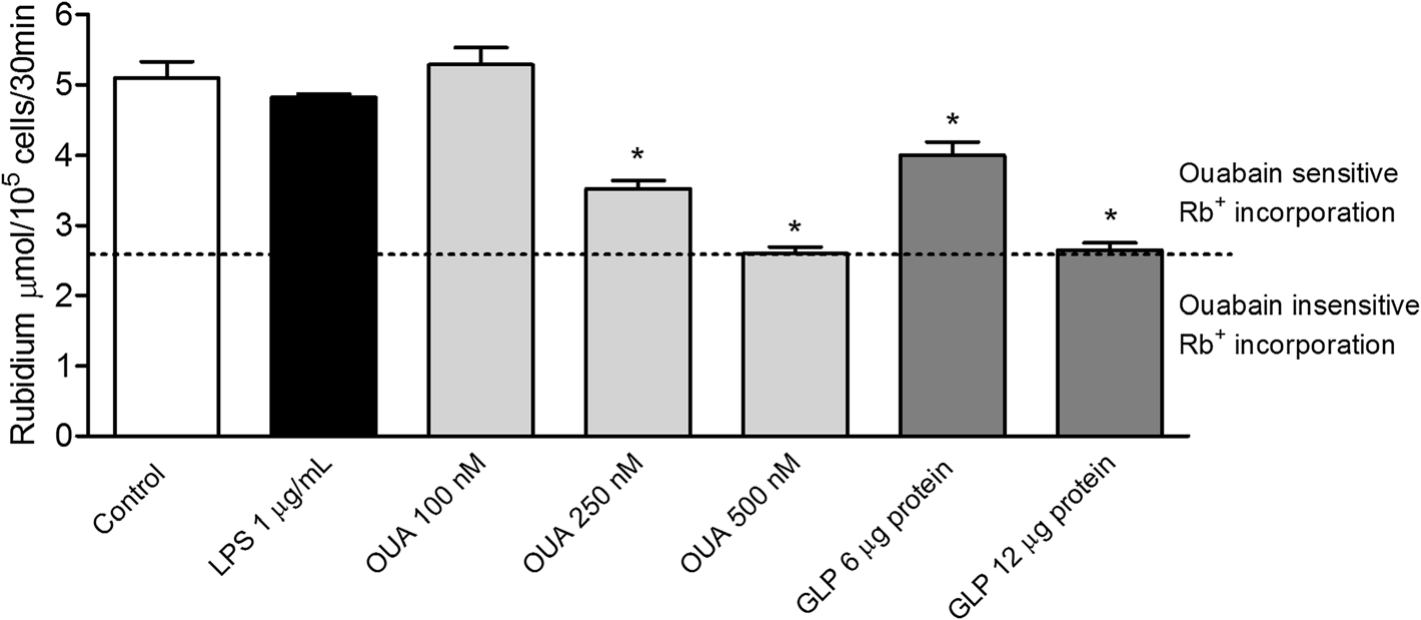
**Inhibition of Rb** ^**+**^ **incorporation into HUVECs by** ***E. coli*** **LPS, ouabain or leptospiral GLP.** The control group was incubated solely with KCl-free Hank’s solution containing non-radioactive Rb^+^. We compared the effects of ouabain (100, 250 and 500 nM), LPS 1 μg/mL and GLP (6 and 12 μg of GLP protein) in the experimental groups. Rb^+^ incorporation in cell cultures after 30 min of treatment was measured by ICP-OES. The results are expressed in μmol Rb^+^ incorporated per hour per 3 × 10^5^ cells. Ouabain-sensitive inhibition of Na/K-ATPase is shown as a difference between Rb^+^ incorporation in the absence and in the presence of ouabain. Ouabain-insensitive Rb^+^ incorporation represents the amount of Rb^+^ entering cells through potassium channels and through passive diffusion. *P < 0.05 compared to the control group.

We showed that GLP inhibited purified Na/KATPase *in vitro* [20]. During leptospirosis, the immune response kills the bacteria, releasing GLP into the bloodstream, where it reaches the lung capillary net. In the present work, ouabain and GLP inhibited Na/K-ATPase in endothelial cells (Figure 8), whereas *E. coli* LPS had no effect.

## Discussion

We used a murine model to study the effects of leptospiral GLP on inflammation [51]. GLP decreased the mRNA levels of Na/K-ATPase β1 *in vitro* [52]. These results revealed that Na/K-ATPase inhibition in alveolar cells is involved in lung edema formation.

Neutrophils are the main cells that migrate to the lung during ARDS. When activated, they release an arsenal of potent molecules that contribute to tissue damage and inflammation [53]. Our data showed neutrophil infiltration after GLP or ouabain injection. Cytokines such as TNF-α and interleukins (mainly IL-1β and IL-6) contribute to the development of ARDS by increasing vascular permeability and organ dysfunction [53]. GLP has also been shown to activate blood mononuclear cells, leading to augmented TNF-α and IL-6 production [54]. Ouabain is known to affect the immune system by modulating cytokine production, and its administration has been shown to induce IL-1 and TNF-α production by mononuclear cells [55]. Our results showed increased lung levels of IL-6, IL-1β, MIP-1α and TNFα in BALF supernatants in GLP- or ouabain-challenged animals. To our knowledge, this is the first report of lung injury induced by intra-tracheal ouabain injection. The chemokine MIP-1α, a chemotactic factor for monocytes [56], was also increased in our model. Therefore, challenge with GLP or ouabain induced the main inflammatory mediators involved in ARDS.

The levels of LTB_4_, a potent chemoattractant for neutrophils [57], increased after GLP or ouabain challenge; therefore, LTB_4_ might be involved in neutrophil migration. The production of PGE_2_ was also elevated after either type of challenge. Recent work has shown that ouabain-induced PGE_2_ production in the murine lung is dependent on cyclooxygenase-2 (COX-2) activation [58]. In this regard, some evidence has suggested that the COX-2/PGE_2_ pathway plays an important role in augmenting the inflammatory immune response during ARDS, as the inhibition of PGE_2_ synthesis prevents edema formation, neutrophil infiltration, pro-inflammatory cytokine production and the expression of adhesion molecules, thereby restoring lung morphology and increasing survival during poly-microbial sepsis [59]. The GLP-induced production of lipid mediators is most likely associated with increased number of lipid bodies, which serve as privileged sites for lipid mediator production [60,61].

Hemorrhage can lead to high mortality rates [62]. Lung hemorrhage can be present in severe leptospirosis [1,6,63,64] but is less frequent in other ARDS etiologies. This hemorrhagic syndrome has been described in leptospirosis patients in China, Korea, Brazil and Nicaragua [65,66]. Animals with leptospirosis infection also display lung hemorrhage [47,67]. We have previously shown that ouabain induces hemorrhagic foci in the lung following local administration [68] and that the hemorrhage is less extensive compared to GLP. GLP induced both lung inflammation and hemorrhage, which contribute to decreased lung function. Lung hemorrhage may have influenced the quantities of cytokines and proteins in the BALF because serum may have flooded the alveoli, increasing the albumin content; therefore, systemic cytokines may have contributed to the total cytokine levels measured in the BALF.

We cannot eliminate the possible presence of a contaminating molecule in our GLP preparation. Nevertheless, when a mouse strain carrying a null TLR4 mutation was compared with the corresponding wild-type strain, we found that GLP did not act through TLR4 activation, which indicates that our GLP preparation was free of Gram(-) LPS. Accordingly, evidence for renal colonization by leptospira inducing a mild renal fibrosis in mice through TLR- and NLR-independent pathways would explain the TLR4-independent effect of GLP [69]. Leptospiral GLP activates the NLRP3 inflammasome protein by down regulating the Na/K-pump [52]. Inflammasome activation is an important step in IL-1β release, and in our study, the IL-1β production in the lung was similar to that induced by GLP and ouabain. Therefore, inflammasome activation may play an important role in the inflammatory events related to leptospirosis.

The signal transduction capacity of Na/K-ATPase occurs through properties different from its function as an ion pump and does not depend on changes in the intracellular Na^+^ and K^+^ concentrations [70]. We showed a new GLP mechanism of action through the activation of p38, whereby Na/K-ATPase activity and lung inflammation triggered by GLP are linked. Therefore, we suggest another mechanism of Na/K-ATPase in lung inflammation beyond its key role in edema formation and/or clearance, strengthening the idea that Na/K-ATPase may be involved in inflammation [71].

Na/K-ATPase has signaling properties [72] that promote intracellular activation [73]. Furthermore, the GLP components oleic acid and ouabain target the Na/K-ATPase in the lung when injected intravenously and cause lung injury [25]. Both GLP and ouabain injected directly into the lung induced similar levels of lung inflammation in SW mice. Furthermore, GLP inhibited Na/K-ATPase in HUVECs and induced inflammatory mediators in both endothelial and lung epithelial cells. Considering Na/K-ATPase as the sole receptor described for ouabain to date, we suggest that GLP may also affect the Na/K-ATPase-dependent activation of signaling cascades.

## Conclusions

GLP and ouabain inhibited Na/K-ATPase in endothelial cells and induced lung injury, as shown by increases in lung inflammation markers and hemorrhage, which also occurs in some leptospirosis patients. To our knowledge, this is the first report showing that GLP induces lung injury. The lung inflammation induced by GLP in our *in vivo* mouse model was not dependent on the activation of TLR4. Although inflammasome activation by low concentrations of intracellular K^+^ surely plays an important role, we showed that GLP activates the p38 pathway, possibly through Na/K-ATPase, resulting in inflammation.

## Abbreviations

GLP: Glycolipoprotein fraction
Na/K-ATPase: Enzyme sodium potassium ATPase
TLR: Toll-like receptor
MAPK: Mitogen-activated protein kinase
NEFA: Nonesterified fatty acids
LLS: Lipopolysaccharide-like substance
LPS: Lipopolysaccharide
ARDS: Acute respiratory distress syndrome
IL: Interleukin
TNF: Tumor necrosis factor
LipL: Leptospiral outer membrane lipoprotein
BALF: Bronchoalveolar lavage fluid
LTB_4_: Leukotriene B_4_
PGE_2_: Prostaglandin E_2_
NLRP3: NLR family, pyrin domain containing 3
ICP-OES: Inductively coupled plasma-optical emission spectrometry
